# Is routine screening for silent pulmonary embolism justified in patients with deep vein thrombosis?

**DOI:** 10.1590/1677-5449.200124

**Published:** 2021-06-25

**Authors:** Marcela Juliano Silva, Cynthia de Almeida Mendes, Sergio Kuzniec, Mariana Krutman, Nelson Wolosker

**Affiliations:** 1 Hospital Israelita Albert Einstein, Cirurgia Vascular, São Paulo, SP, Brasil.

**Keywords:** venous thrombosis, vena cava filters, pulmonary embolism, embolism and thrombosis, anticoagulants, trombose, veias cavas, embolia, embolia e trombose, anticoagulantes

## Abstract

The incidence of asymptomatic pulmonary embolism (PE) exceeds 70% in patients with deep venous thrombosis (DVT), even in cases of distal deep vein thrombosis. We report the case of a patient with a diagnosis of DVT in the lower left limb associated with asymptomatic PE who presented late symptoms due to this same PE. The absence of acute symptoms and the late onset of symptoms could have provoked doubts about the most appropriate treatment, resulting in unnecessary interventions, if pulmonary embolism had not already been diagnosed with tomography. In the present case, we demonstrate that computed tomography angiography conducted at the time of DVT diagnosis accurately diagnosed PE and prevented any misinterpretation of recurrent DVT in a patient already being medicated, which could have been mistakenly interpreted as demonstrating failure of anticoagulant therapy. Such a situation could lead to unnecessary intervention to fit an inferior vena cava filter. We cannot suggest that a classic medical conduct should be reformulated simply on the basis of a case report. However, we would be remiss not to suggest that well-designed studies should be carried out in the future to assess the need for this examination in the acute phase.

## Resumo

A ocorrência de embolia pulmonar assintomática em pacientes com trombose venosa profunda tem uma incidência que excede 70%, mesmo nos casos de trombose venosa profunda distal. Relatamos o caso de um paciente com diagnóstico de trombose venosa profunda no membro inferior esquerdo associado a embolia pulmonar assintomática, que apresentou sintomas tardios devido a essa mesma embolia. A ausência de sintomas agudos e o surgimento de sintomas tardios pode suscitar dúvidas quanto ao tratamento mais adequado e resultar em intervenções desnecessárias, se o diagnóstico tomográfico de embolia pulmonar não tiver sido feito anteriormente. No presente caso, demonstramos que uma angiotomografia realizada no momento do diagnóstico de trombose venosa profunda detectou a embolia pulmonar e evitou uma interpretação incorreta de um evento trombótico recorrente na vigência de anticoagulação, o que por engano demonstraria uma falha na terapia anticoagulante. Essa situação pode levar a intervenções desnecessárias, como o implante de filtro de veia cava inferior. Entendemos que apenas um relato de caso não deve mudar uma conduta médica já estabelecida; no entanto, fomenta a discussão e estimula estudos que avaliem a necessidade de um exame diagnóstico pulmonar no momento do diagnóstico de trombose venosa profunda.

## INTRODUCTION

Occurrence of asymptomatic pulmonary embolism (PE) in patients with deep venous thrombosis (DVT) was described by Kistner et al.^1^^,^^2^ in the 1970s and its reported incidence exceeds 70%, even in cases of distal DVT.

In general, asymptomatic PE has no clinical consequences and does not lead to treatment changes. There is therefore a discussion on whether there is any utility in conducting diagnostic tests to investigate it.^3^

Finding asymptomatic PE is simple and safe these days, especially with pulmonary computed tomography angiography (CTA). This imaging exam can be safely performed in patients with creatinine clearance above 30 mL/min and who are not allergic to iodine. It accurately identifies thrombi in the pulmonary artery tree and provides information on their location.^4^

Pulmonary embolism occurs after hospital discharge in up to 3.5% of fully anticoagulated individuals. This clinical manifestation suggests therapeutic failure of anticoagulation and can be an indication for vena cava filter implantation, which is an invasive procedure performed to avoid PE in well-selected cases, but which can lead to short and long-term complications.^5^^,^^6^

We report the case of a patient with a diagnosis of DVT in the left lower limb associated with asymptomatic PE diagnosed by CTA during hospitalization who later presented symptoms due to this same PE. The Research Ethics Committee approved this study (decision number 4.791.994).

## PART I – CLINICAL SITUATION

A 45-year-old male presented with pain in the left leg with two days’ duration. He had a personal history of gastrocnemius vein thrombosis in the left lower limb five years earlier, associated with long-haul air travel, and extensive thrombophlebitis in the right upper limb after using a peripheral venous access catheter for treatment of a short-duration gastrointestinal event. At the time of the first DVT episode, a heterozygous prothrombin mutation and low protein S levels (41%, Reference Value: 55-140%) were identified. The patient remained asymptomatic for five years using prophylactic enoxaparin for long-haul flights.

**Figure 1:**
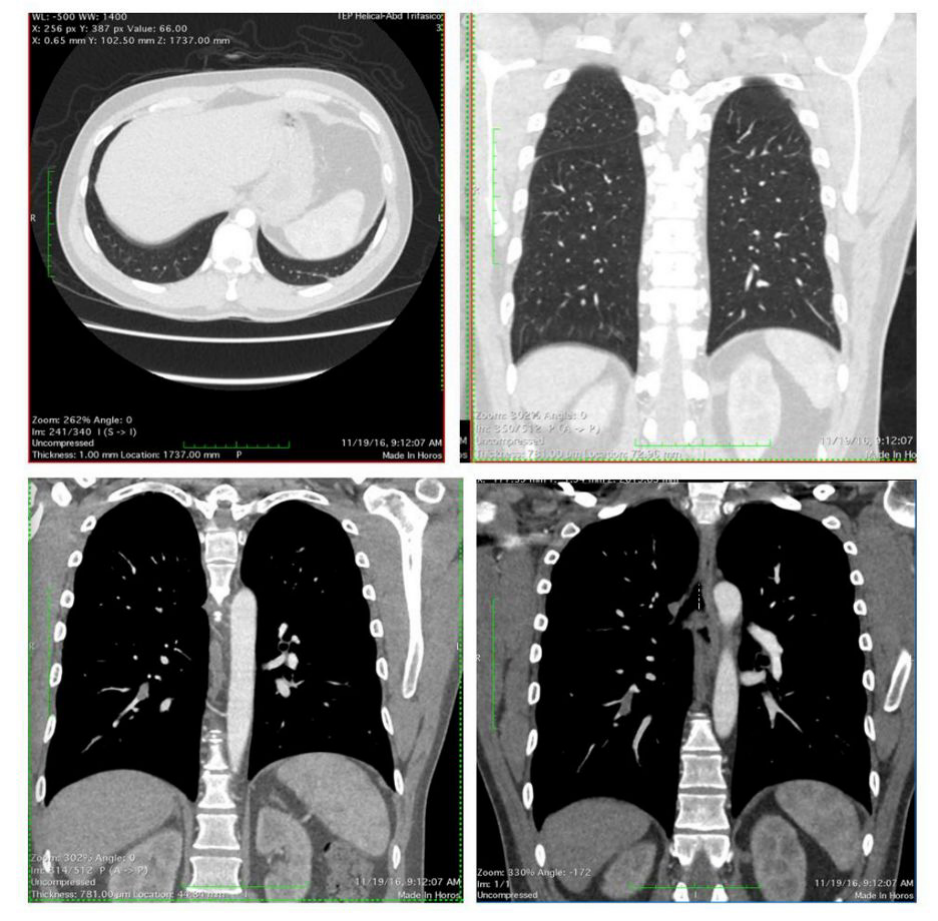
CTA diagnosing segmental and sub-segmental thrombus in the right lung.

In the present event, no edema or muscle swelling was observed on physical examination, but the calf musculature was painful on palpation. Doppler ultrasonography (USG) was performed and demonstrated acute thrombosis of the popliteal and medial gastrocnemius veins. The patient did not complain of coughing, dyspnea, or chest pains. Nevertheless, CTA was performed by decision of our team and showed segmental and subsegmental thrombi in the right lung (Figure 1), which was thus diagnosed as PE. The patient was admitted for treatment with parenteral anticoagulation with enoxaparin 1mg/kg, bid., bed rest and pain killers. There was progressive improvement in pain and the patient was discharged on the fourth day of hospitalization on oral anticoagulation with rivaroxaban at a dose of 15 mg bid., to be maintained for 21 days, when it would be changed to a dosage of 20 mg daily.

**Figure 2:**
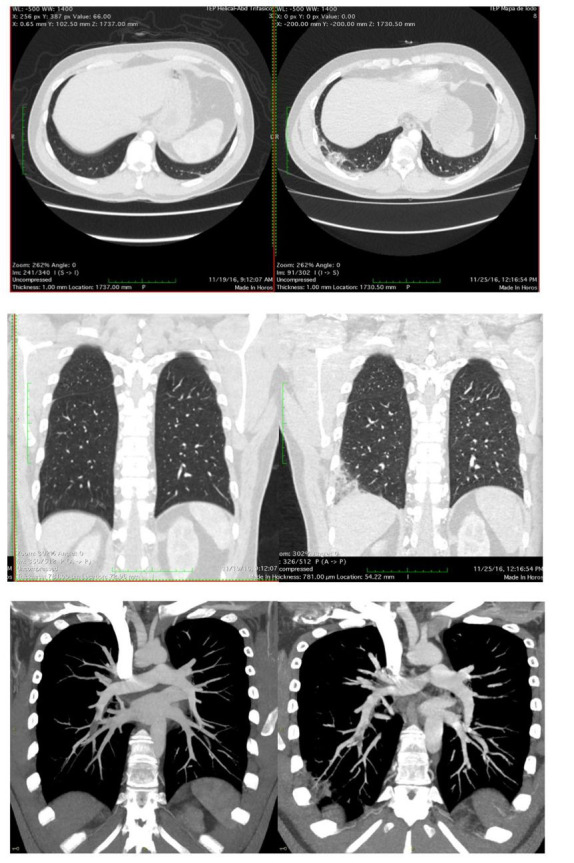
Comparison between the CTAs in the asymptomatic phase (left) and after pulmonary infarction pain (right).

After five days, the patient complained of intense pain in the right posterior thoracic region and respiratory discomfort for two hours, with no signs of respiratory failure. He returned to the emergency department and underwent another chest CTA, which showed partial recanalization of the thrombi identified in the previous exam, and areas of pulmonary infarction, in the lung segment corresponding to the area of the embolism, coincident with the location of the chest pain, with no evidence of new emboli (Figure 2).

## PART II – WHAT WAS DONE

Having diagnosed pulmonary infarction without new embolism, we chose to maintain rivaroxaban and analgesia. There was good clinical progress and a 4-month follow-up CTA showed complete recanalization of the pulmonary arterial tree and resolution of pulmonary infarction. Since then, no new episodes of venous thromboembolism have occurred and the patient remains on anticoagulation. He has been asymptomatic for the last four years.

## DISCUSSION

In a 2016 study conducted by Krutman et al.^2^^,^^7^ with a group of 52 patients with acute lower limb DVT without respiratory symptoms, presence of PE was demonstrated in 72.7% of patients with proximal DVT and in 73.7% of those with distal DVT. In most cases (68%), these emboli involved larger segments of the pulmonary arteries and in 32% they affected subsegmental arteries. In common with that study, the patient described in this case report had acute popliteal vein and gastrocnemius veins thrombosis and did not have any respiratory symptoms. Nevertheless, he had PE with segmental and subsegmental thrombi in the right lung.

Some studies suggest that untreated PE is associated with in-hospital mortality of 30% or more. This risk is even higher when the patient has pulmonary, oncological, or cardiological diseases.^1^ In patients who survive the acute phase of asymptomatic PE, late mortality can occur due to related complications, such as chronic pulmonary hypertension, dead spaces, and loss of the alveolar-capillary O_2_ gradient.^1^^,^^4^ We therefore discuss whether or not it is necessary to perform CTA scans on patients with DVT in the lower limbs, even if they do not present symptoms suggestive of PE.^1^

García-Fuster et al.^4^ evaluated 103 inpatients with DVT. They performed CTA on all asymptomatic patients and found a 66% incidence of silent PE. However, in their conclusions they suggest that silent PE did not imply immediate or late morbidity in their 3-year follow-up and neither did it require an additional treatment strategy.

Although the clinical importance of asymptomatic PE remains unclear, there is evidence indicating that presence of a peripheral pulmonary embolus is a predictor of recurrence of venous thromboembolism (VTE) with the potential to develop new, more severe or fatal events. It is estimated that the recurrence rate of VTE after 1 year is 11% in patients with asymptomatic PE.^2^^,^^7^

In 2001, Ruiz et al.^8^ demonstrated that six out of 200 patients with asymptomatic DVT and PE (identified by pulmonary scintigraphy) developed symptoms within seven days of follow-up, without presenting new perfusion defects in additional lung mapping. Moreover, seven patients presented recurrent PE with new perfusion defects. For these patients, it was necessary to proceed to vena cava filter implantation.

In the present case, the CTA performed at the time of DVT diagnosis accurately diagnosed PE and prevented any misinterpretation of recurrent PE, which could have been mistakenly interpreted as demonstrating a failure of anticoagulant therapy. Such a situation could lead to unnecessary intervention to implant an inferior vena cava filter, for example, according to ACCP guidelines.^9^

CTA is considered a noninvasive examination, but there is a risk of contrast-induced nephropathy and radiation exposure, which should be taken into account.^1^^,^^2^ Alternatively, pulmonary ventilation and perfusion scintigraphy may be performed.^2^ When taking the decision on whether to perform CTA or not, we should keep in mind that silent PE does not cause clinical consequences for most patients and its diagnosis does not change the treatment.^4^ The costs of performing a routine supplementary examination should also be considered.

On the other hand, in patients with indications for full anticoagulation who present therapeutic failure or bleeding that contraindicate maintenance of anticoagulation, inferior vena cava filter implantation is indicated to avoid PE.^10^ However, this procedure is not free from complications and involves considerable cost. In 2010, Spencer et al. evaluated 1547 patients with DVT and found that 25% of filters were inserted inappropriately.^11^ Furthermore, according to the PREPIC study, 13% of vena cava filters present thrombosis, accounting for 45.6% of the causes of recurrent DVT.^12^

It is also necessary to discuss the decision to use rivaroxaban in a patient with thrombophilia. Skelley et al. and Elsebaie et al. have both stated that the direct oral anticoagulants are suitable alternatives to vitamin K antagonists (VKA) and that rates of VTE recurrence and bleeding events were low and comparable with patients with thrombophilia taking VKA. Further studies are still needed for confirmation and to provide additional evidence.^13^^,^^14^

In our case, prior identification of PE in an asymptomatic patient with DVT, diagnosed by CTA, was decisive in the subsequent clinical management of the case. Without the initial exam, the symptomatic episode would likely have been interpreted as PE in a patient on full anticoagulation, after 7 days of clinical treatment. It would then have been reasonable to assume failure of the clinical therapy and a need for inferior vena cava filter implantation, besides changing the anticoagulation method. The information provided by the CTA performed when asymptomatic was decisive to demonstrate that there had been a PE before clinical treatment was implemented and that the symptoms were due to focal pulmonary ischemic pain and to the clinical course of this single and initial embolic event.

DVT guidelines suggest that patients with DVT without pulmonary symptoms do not need a radiological investigation to look for PE. In this case, the patient underwent CTA and benefited from early diagnosis of an asymptomatic PE. Obviously, we cannot suggest that a classic medical conduct should be reformulated simply on the basis of a case report. However, we would be remiss not to suggest that well-designed studies should be carried out in the future to assess the need for this examination in the acute phase.
